# Large scale genomic analysis of 3067 SARS-CoV-2 genomes reveals a clonal geo-distribution and a rich genetic variations of hotspots mutations

**DOI:** 10.1371/journal.pone.0240345

**Published:** 2020-11-10

**Authors:** Meriem Laamarti, Tarek Alouane, Souad Kartti, M. W. Chemao-Elfihri, Mohammed Hakmi, Abdelomunim Essabbar, Mohamed Laamarti, Haitam Hlali, Houda Bendani, Nassma Boumajdi, Oussama Benhrif, Loubna Allam, Naima El Hafidi, Rachid El Jaoudi, Imane Allali, Nabila Marchoudi, Jamal Fekkak, Houda Benrahma, Chakib Nejjari, Saaid Amzazi, Lahcen Belyamani, Azeddine Ibrahimi

**Affiliations:** 1 Medical Biotechnology Laboratory (MedBiotech), Bioinova Research Center, Rabat Medical & Pharmacy School, Mohammed Vth University, Rabat, Morocco; 2 Laboratory of Human Pathologies Biology, Department of Biology, Faculty of Sciences, and Genomic Center of Human Pathologies, Faculty of Medicine and Pharmacy, Mohammed V University, Rabat, Morocco; 3 Anoual Laboratory of Radio-Immuno Analysis, Casablanca, Morocco; 4 Faculty of Medicine, Mohammed VI University of Health Sciences (UM6SS), Casablanca, Morocco; 5 International School of Public Health, Mohammed VI University of Health Sciences (UM6SS), Casablanca, Morocco; 6 Emergency Department, Military Hospital Mohammed V, Rabat Medical & Pharmacy School, Mohammed Vth University, Rabat, Morocco; Duke University, UNITED STATES

## Abstract

In late December 2019, an emerging viral infection COVID-19 was identified in Wuhan, China, and became a global pandemic. Characterization of the genetic variants of SARS-CoV-2 is crucial in following and evaluating it spread across countries. In this study, we collected and analyzed 3,067 SARS-CoV-2 genomes isolated from 55 countries during the first three months after the onset of this virus. Using comparative genomics analysis, we traced the profiles of the whole-genome mutations and compared the frequency of each mutation in the studied population. The accumulation of mutations during the epidemic period with their geographic locations was also monitored. The results showed 782 variants sites, of which 512 (65.47%) had a non-synonymous effect. Frequencies of mutated alleles revealed the presence of 68 recurrent mutations, including ten hotspot non-synonymous mutations with a prevalence higher than 0.10 in this population and distributed in six SARS-CoV-2 genes. The distribution of these recurrent mutations on the world map revealed that certain genotypes are specific to geographic locations. We also identified co-occurring mutations resulting in the presence of several haplotypes. Moreover, evolution over time has shown a mechanism of mutation co-accumulation which might affect the severity and spread of the SARS-CoV-2. The phylogentic analysis identified two major Clades C1 and C2 harboring mutations L3606F and G614D, respectively and both emerging for the first time in China. On the other hand, analysis of the selective pressure revealed the presence of negatively selected residues that could be taken into considerations as therapeutic targets. We have also created an inclusive unified database (http://covid-19.medbiotech.ma) that lists all of the genetic variants of the SARS-CoV-2 genomes found in this study with phylogeographic analysis around the world.

## Introduction

The recent emergence of the novel, human pathogen Severe Acute Respiratory Syndrome Coronavirus 2 (SARS-CoV-2) in China with its rapid international spread poses a global health emergency. On March 11, 2020, the World Health Organization (WHO) publicly announced the SARS-CoV-2 epidemic as a global pandemic. As of Mai 01, 2020, the COVID-19 pandemic had affected more than 200 countries and territories, with more than 3,175,207 confirmed cases and 224,172 deaths [1].

The new SARS-CoV-2 coronavirus is an enveloped positive-sense single-stranded RNA virus belonging to a large family named coronavirus which have been classified under three groups two of them are responsible for infections in mammals [2], such us: bat SARS-CoV-like; Middle East respiratory syndrome coronavirus (MERS-CoV). Many recent studies have suggested that SARS-CoV-2 was diverged from bat SARS-CoV-like [3, 4].

The size of the SARS-CoV2 genome is approximately 30 kb and its genomic structure has followed the characteristics of known genes of Coronavirus; the polyprotein orf1ab also known as the polyprotein replicase covers more than 2 thirds of the total genome size while the rest contains structural proteins, including spike protein, membrane protein, envelope protein and nucleocapsid protein. In addition to six ORFs (ORF3a, ORF6, ORF7a, ORF7b, ORF8 and ORF10) predicted as hypothetical proteins with no associated function [5].

Characterization of viral mutations can provide valuable information for assessing the mechanisms linked to pathogenesis, immune evasion and viral drug resistance. A previous study [6] based on an analysis of 103 genomes of SARS-CoV-2 indicates that this virus has evolved into two main types, type L being more widespread than type S, and type S representing the ancestral version. Another study [7] conducted on 32 genomes of strains sampled from China, Thailand and the United States between December 24, 2019 and January 23, 2020 suggested increasing tree-like signals from 0 to 8.2%, 18.2% and 25, 4% over time, which may indicate an increase in the genetic diversity of SARS-CoV-2 in human hosts.

Therefore, the analysis of mutations and monitoring of the evolutionary capacity of SARS-CoV-2 over time-based on a large population is necessary. In this study, we characterized the genetic variants in 3067 SARS-CoV-2 genomes for a detailed understanding of their genetic diversity and to monitor the accumulation of mutations over time with particular focus on the geographic distribution of recurrent mutations. On the other hand, we established selective pressure analysis to predict negatively selected residues which could be useful for the design of therapeutic targets. We have also created a database to share, exploit and research knowledge of genetic variants to facilitate SARS-CoV-2 comparison for thescientific community.

## Materials and methods

### Data collection and variant calling analysis

3067 sequences of SARS-CoV-2 were collected from the GISAID EpiCovTM (update: 02-04-2020) and NCBI (update: 20-03-2020) databases. Only complete genomes were used in this study (**S1 Table**). Genomes were mapped to the reference sequence Wuhan-Hu-1/2019 (NC_045512) using Minimap v2.12-r847-dirty [8]. The BAM files were sorted by SAMtools sort [9], then used to call the genetic variants in variant call format (VCF) by SAMtools mpileup [9] and bcftools v1.8 [9]. The final call set of the 3067 genomes, was annotated and their impact was predicted using SnpEff v 4.3t [10]. First, the SnpEff databases were built locally using annotations of the reference genome NC_045512.2 obtained in GFF format from the NCBI database. Then, the SnpEff database was used to annotate single nucleotide polymorphisms (SNPs) and insertions/deletions (InDels) with putative functional effects according to the categories defined in the SnpEff manual (http://snpeff.sourceforge.net/SnpEff_manual.html).

### Phylogentic analysis and geodistribution

The downloaded full-length genome sequences of coronaviruses isolated from different hosts from public databases were subjected to multiple sequence alignments using MAFFT v7.4 [11]. Maximum-likelihood trees were inferred with IQ-TREE v1.5.5 [12] under the GTR model using NextStrain tools. Heatmap for correlation analysis was performed on countries and hotspots mutations using CustVis with default settings rows scaling = variance scaling, PCA method = SVD with imputation, clustering distance for rows = correlation clustering, the method for rows = average, tree ordering for rows = tightest cluster first [13].

### Selective pressure and modelling

We used Hyphy v2.5.8 [14] to estimate synonymous and non-synonymous ratio dN / dS (ω). Two datasets of 191 and 433 for orf1ab and genes respectively were retrieved from Genbank (http://www.ncbi.nlm.nih.gov/genbank/). After deletion of duplicated and cleaning the sequences, only 91 and 39 for orf1ab and spike proteins, respectively, were used for the analysis (**S2 Table**). The selected nucleotide sequences of each dataset were aligned using Clustalw codon-by-codon and the phylogenetic tree was obtained using ML (maximum likelihood) available in MEGA X [15]. For this analysis, four Hyphy's methods were used to study site-specific selection: SLAC (Single-Likelihood Ancestor Counting) [16], FEL (Fixed Effects Likelihood) [16], FUBAR (Fast, Unconstrained Bayesian AppRoximation) [17] and MEME (Mixed Effects Model of Evolution) [18]. For all the methods, values supplied by default were used for statistical confirmation and the overall ω value was calculated according to ML trees under General time reversible model (GTR model). The CI- TASSER generated models (https://zhanglab.ccmb.med.umich.edu/COVID-19/) of nonstructural proteins (nsp3, nsp4, nsp6, nsp12, nsp13, nsp14 and nsp16 of orf1ab were used to highlight the sites under selective pressure on the protein. On the other hand, the cryo-EM structure with PDB id 6VSB was used as a model for the spike protein in its prefusion conformation. Structure visualization and image rendering were performed in PyMOL 2.3 (Schrodinger LLC).

### Pangenome construction

115 proteomes of the genus *Betacorononavirus* were obtained from the NCBI database (update: 20-03-2020), of which 83 genomes belonged to the SARS-CoV-2 species and the rest distributed to other species of the same genus publicly available (**S3 Table**). These proteomes were used for the construction of pangenome at the inter-specific scale of *Betacoronavirus* and intra-genomic of SARS-CoV-2. The strategy of best reciprocal BLAST results [19] was implemented to identify all of the orthologous genes using Proteinortho v6.0b [20]. Proteins with an identity above 60% and sequence coverage above 75% with an e-value threshold below 1e-5 were used to be considered as significant hits.

## Results

### SARS-CoV-2 genomes used in this study

**Fig 1:**
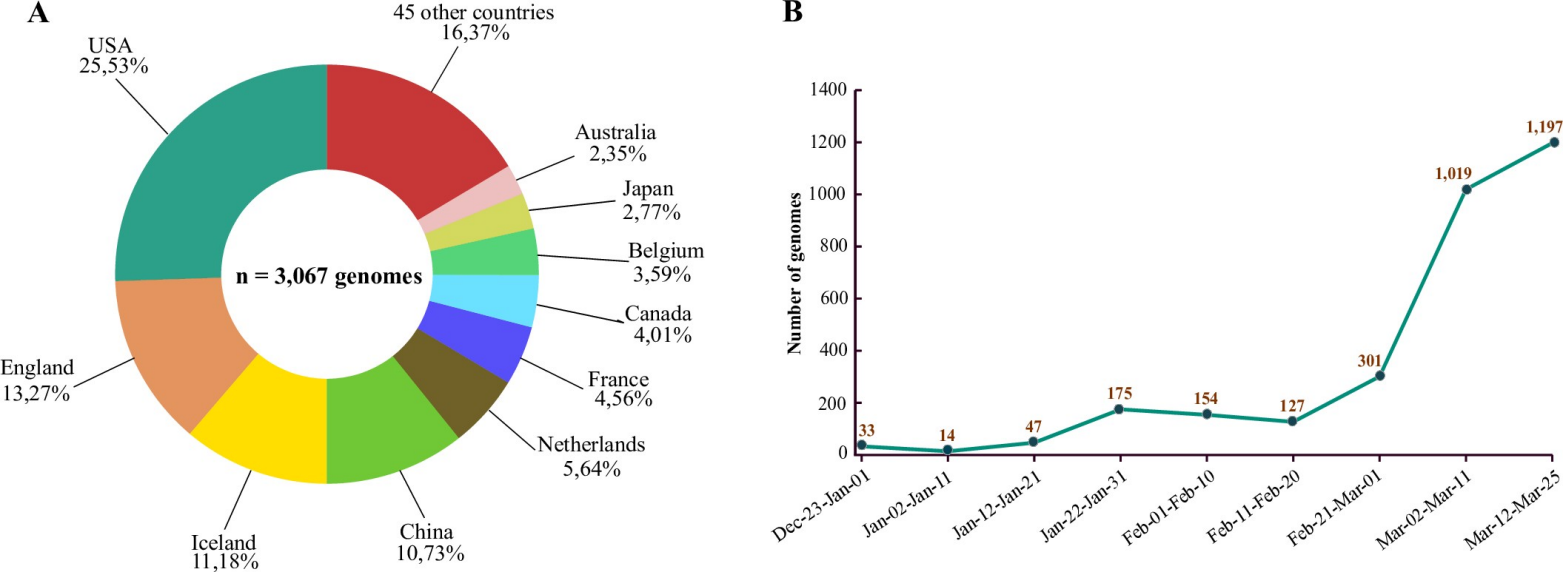
Distribution of the 3,067 genomes used in this study by country and date of isolation. A) The pie chart represents the percentage of genomes used in this study according to their geographic origins. The colors indicate different countries. B) Number of genomes of complete pathogens, distributed over a period of 3 months from the end of December to the end of March.

In this study, we used 3,067 SARS-CoV2 complete genomes collected from GISAID EpiCovTM (update: 02-04-2020) and NCBI (update: 20-03-2020) databases. These strains were isolated from 55 countries (**Fig 1A**). The most represented origin was American strains with 783 (25.53%), followed by strains from England, Iceland, and China with 407 (13.27%), 343 (11.18%), 329 (10.73%), respectively. The date of isolation was during the first three months after the appearance of the SARS-CoV-2 virus, from December 24, 2019, to March 25, 2020 (**Fig 1B**). Likewise, about two-thirds of these strains collected in this work were isolated during March.

### Mutational frequency analysis revealed a diversity of genetic variants in six SARS-Cov-2 genes

**Fig 2:**
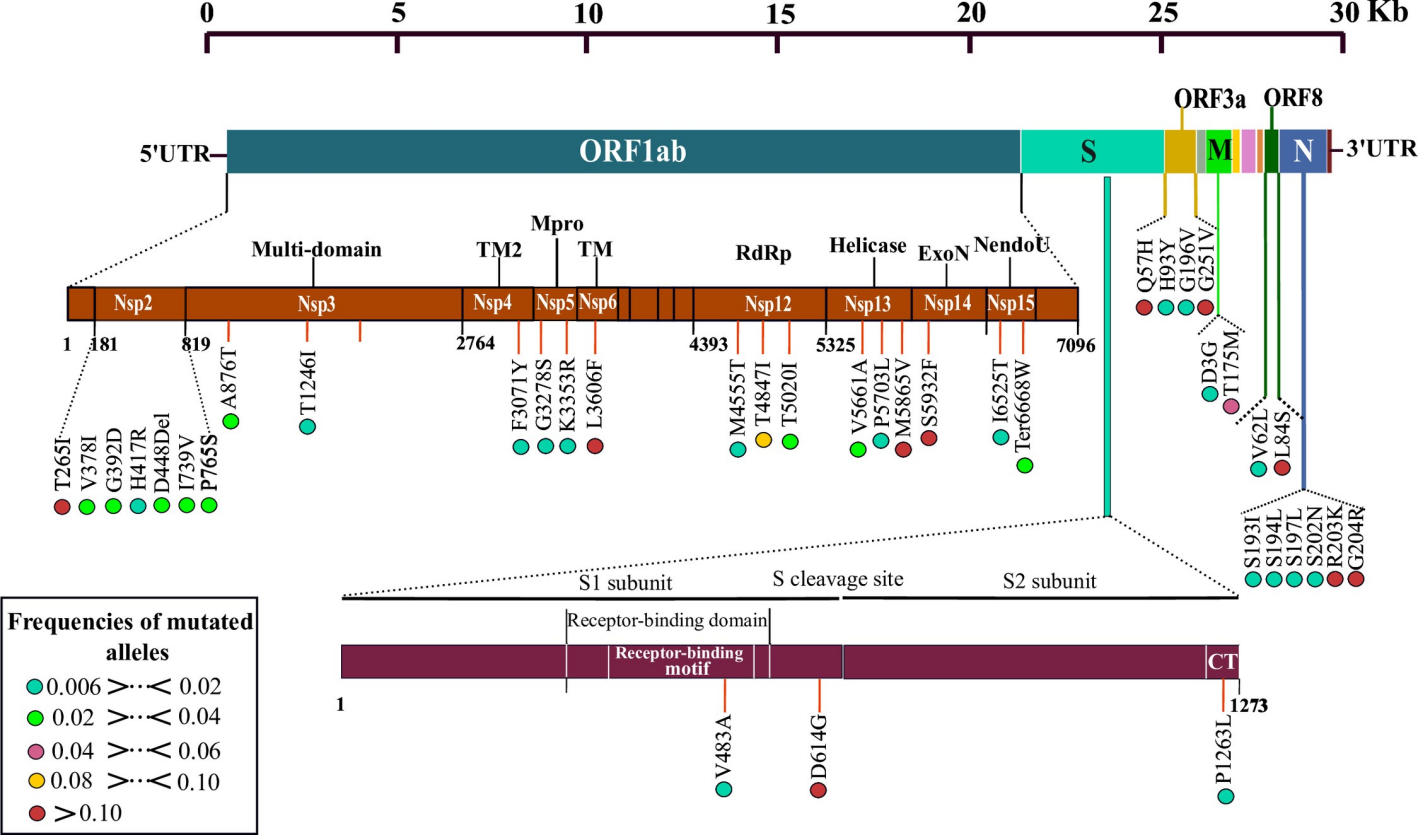
Schematic representation illustrating the distribution of recurrent non-synonymous mutations along the SARS-CoV-2 genome. The brown and garnet diagrams illustrate the non-structural proteins (nsp1 to nsp 16) of the orf1ab protein and the two subunits of the spike protein, respectively. Recurrent mutations represented by vertical lines. The frequency of each mutation in the population is presented by color coded circles. Abbreviations: S, spike; E, enveloppe; M, membrane protein; N, nucleocapsid protein; CT, Cytoplasmic chail.

A total of 782 variant sites were detected compared to the Wuhan-Hu-1/2019 reference sequence, of which 65.98% having a non-synonymous effect, 28.39% synonymous mutations, and 5.63% are distributed in the regions intergenic. Mutational frequency analysis revealed the presence of 68 mutations with a frequency greater than 0.06% of the total genomes, which corresponds to at least 20/3067 genomes. Focusing on non-synonymous mutations (freq> 0.06% of the total genomes), 38 were identified and distributed in six SARS-CoV-2 genes at variable frequencies (**Fig 2**). Among them, the ORF1ab polyprotein harbored 22 non-synonymous mutations: seven in nsp2 (T265I, V378I, G392D, H417R, I739V, P765S, and D448Del) three in nsp12-RdRp (M4555T, T4847I and T5020I), three in nsp13-nsp13 (V5661A, P5703L, and M5865V), two in nsp3-multi-domains (A876T and T1246I), two in nsp5-main proteinase (G3278S and K3353R), two in nsp15-EndoRNAse (I6525T, Ter6668W), and one in each of three proteins; nsp6-transmembrane domain (L3606F), nsp4-transmembrane domain-2 (F3071Y), and nsp14-exonuclease (S5932F).

Likewise, the spike protein harbored three non-synonymous mutations, including V483A in the receptor-binding domain (RBD). The remainder was found in the core phosphoprotein (S193I, S194L, S197L, S202N, R203K, and G204R), membrane glycoprotein (D3G, T175M), ORF3a (Q57H, H93Y, G196V, and G251S V62) and ORF62884).

### Identification of ten hyper-variable genomic hotspot in SARS-CoV-2 genomes

Interestingly, among all recurrent mutations, ten were found as hotspot mutations with a frequency greater than 0.10 in this study population (**Fig 2**). The most represented was D614G mutation at spike protein with 43.46% (n = 1.333) of the genomes, the second was L84S (at ORF8) found in 23.21% (n = 712). Thus, the gene coding for orf1ab had four mutations hotspots, including S5932F of nsp14-exonuclease, M5865V of nsp13 helicase L3606F of nsp6 transmembrane domain and T265I of nsp2 found with 17.02%, 16.56%, 14.38% and 10.66% of the total genomes, respectively. For the four other hotspot mutations were distributed in ORF3a (Q57H and G251V) and nucleocapsid phosphoprotein (R203K and G204R).

### Geographical distribution and origin of mutations worldwide

**Fig 3:**
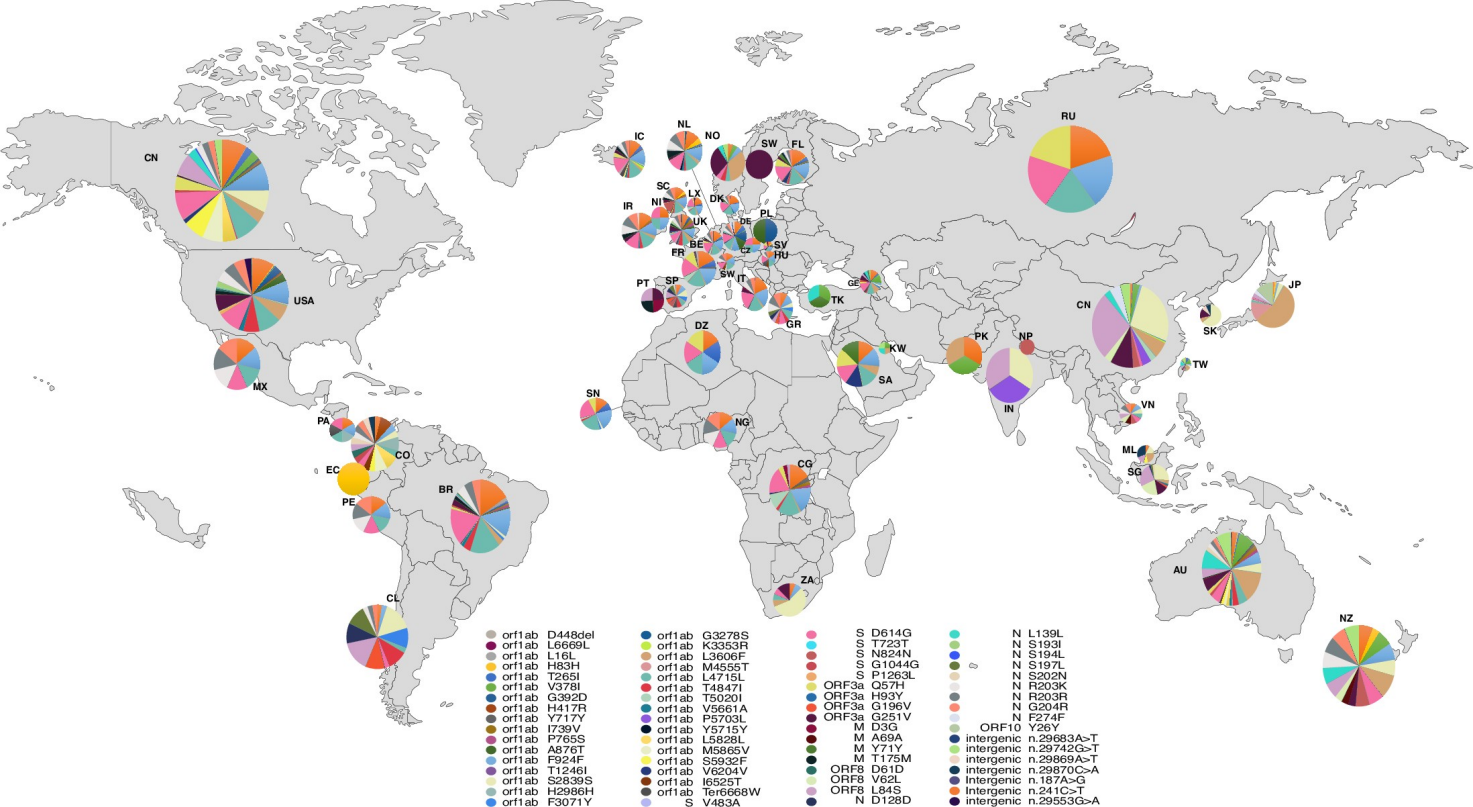
Map showing geographical distribution of recurrent mutation in the studied population worldwide. The pie charts show the relative frequencies of haplotype for each population. The haplotypes are color coded as shown in the key. The double-digit represent countries' two letters code. The circle's size was randomly generated with no association with the number of genomes in each country. Abbreviations: S, spike; E, enveloppe; M, membrane protein; N, nucleocapsid protein.

3067 genomes were dispersed in different countries with different genotype profiles. We performed a geo-referencing mutation analysis to identify region-specific loci. China and the USA were the countries with the highest number of mutations 301 and 296 correspondings, respectively to 38,19% and 37,56% of the total number of mutations. These mutations include 140 (17,76%) and 229 (29%) singleton mutations specific to China and USA genomes, respectively, which is mainly due to the high number of genomes available in these countries. However, England contains more than 300 genomes and harbored only 116 mutations only 23 of theme were singleton mutations. Data normalization shows that the hight mutational rate was observed in New Zealand followed by Malaysia and Vietnam, respectively, While the number of singleton mutations per genome was higher in Malaysia (20,16%), China (5,2%), and the USA (2%), respectively. It is interesting to note that among the 55 countries, 21 harbored singleton mutations. **S4 and S5 Tables** illustrates the detailed singleton mutations found in these countries. The majority of the genomes analyzed carried more than one mutation. However, among the recurrent non-synonymous, synonymous, deletion and intergenic mutations, we found G251V (in ORF3a), and S5932F (in ORF1ab) present on all continents except Africa (**Fig 3**). While F924F, L4715L (in orf1ab), D614G (in spike) appeared in all strains except those from Asia. In Algeria, the genomes harbored mutations very similar to those in Europe, including two recurrent mutations T265I and Q57H of the ORF3a. Likewise, the European and Dutch genomes also shared ten recurrent mutations. On the other hand, continent-specific mutations have also been observed, for example in America, we found seven mutations shared in almost all genomes. Besides, two mutations at positions 28117 and 28144 were shared by the Asian genomes, while four different positions 1059, 14408, 23403, 25563 and 1397, 11083, 28674, 29742 were shared by African and Australian genomes (S1 Table). The majority of these mutations are considered to be transition mutations with a high ratio of A substituted by G. The genome variability was more visible in China and USA than in the rest of the world.

SARS-CoV-2 genomes also harbored three co-occurrent mutations R203K, R203R and G204R in the N protein and were present in all continents except Africa and Asia (besides Taiwan).

### Evolution of mutations over time

**Fig 4:**
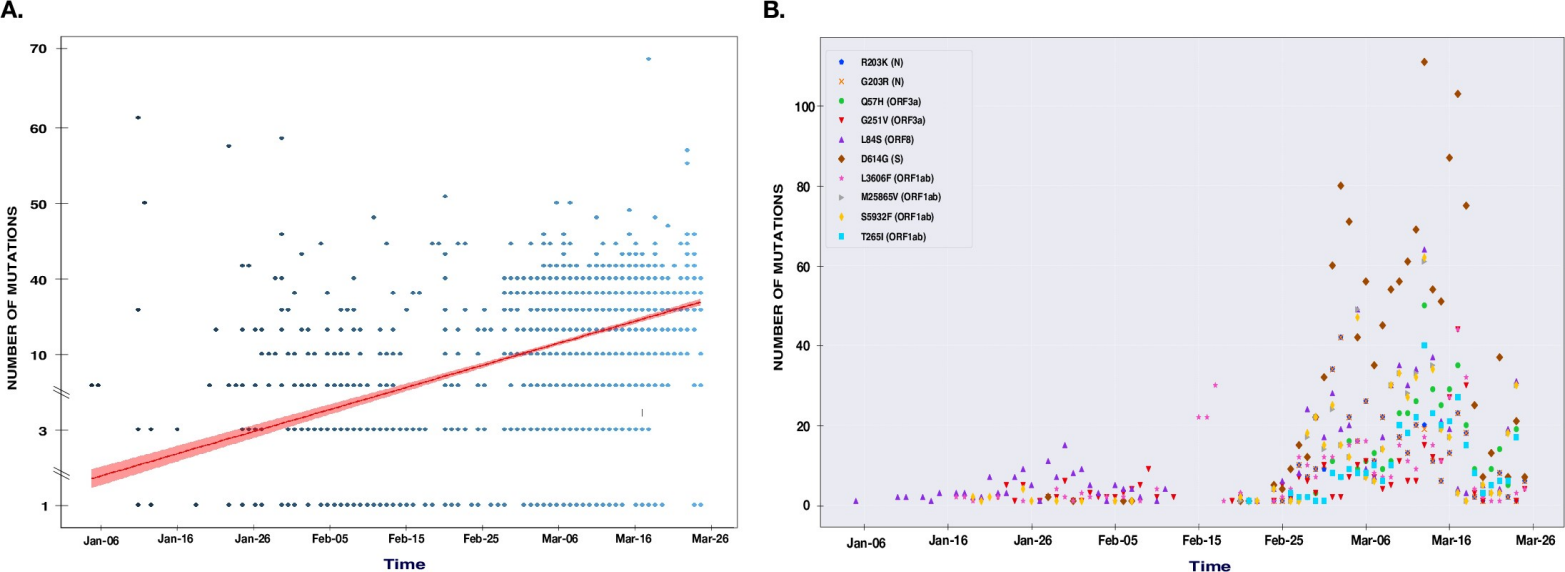
The graph represents substitutions accumulation in a three months period. A) The accumulation of mutations increases linearly with time. The dots represent the number of mutations in each genome. All substitutions have been included: non-synonymous, synonymous and intergenic mutations. B) The distribution and accumulation of Hot spot mutations over time.

**Fig 5:**
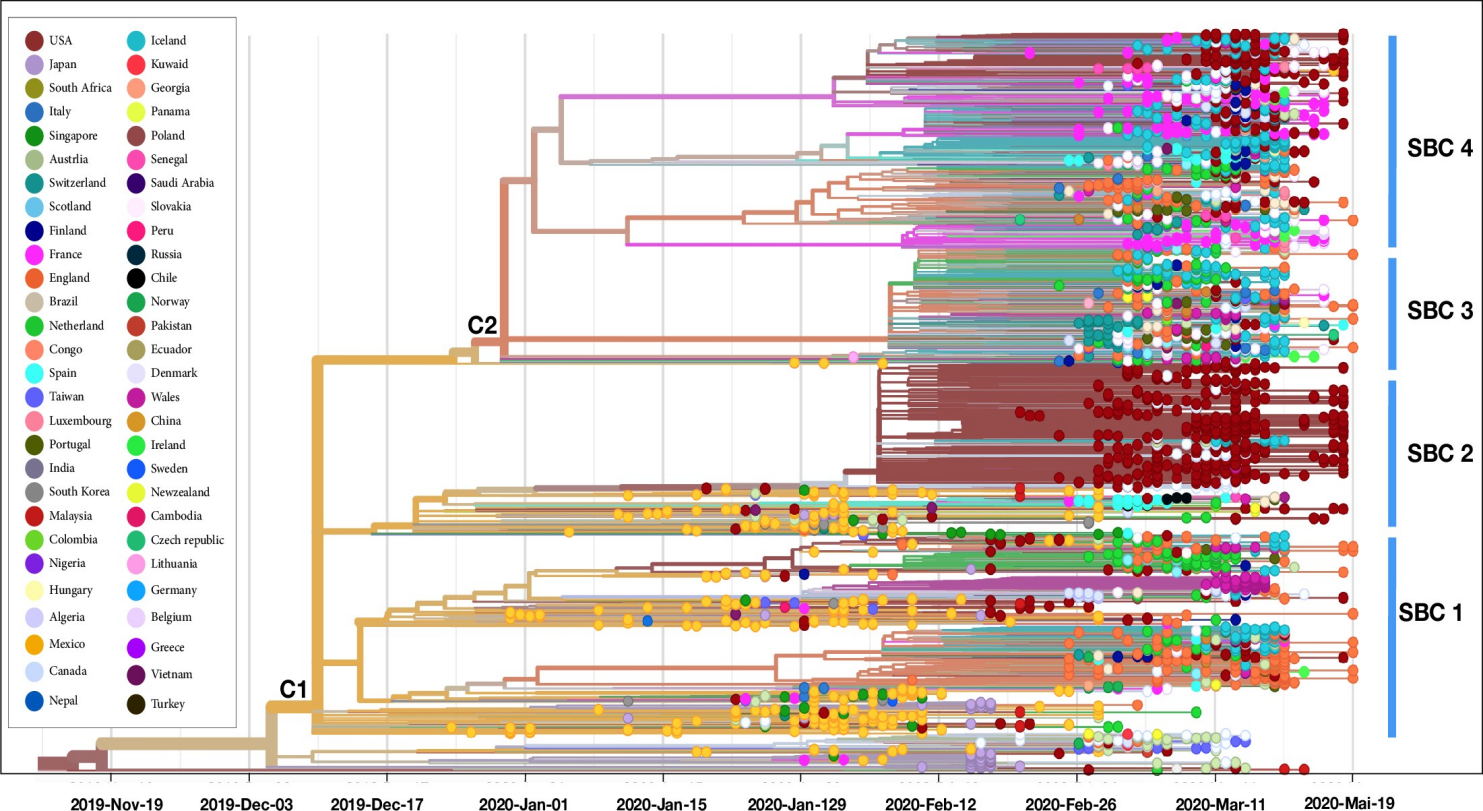
Phylogenetic analysis of 3067 SARS-CoV 2 genomes grouped according to the country of origin. The length of the branches represents the distance in time.

We selected the genomes of the SARS-CoV-2 virus during the first three months after the emergence of this virus (December 24 to March 25). We have noticed that the mutations have accumulated at a relatively constant rate (**Fig 4A**). The strains selected at the end of March showed a slight increase in the accumulation of mutations with an average of 11.34 mutations per genome, compared to the gnomes of February, December and January with an average number of mutations of 9.26, 10.59 and 10.34 respectively. The linear curve in Fig 5 suggests a continuous accumulation of single SNPs in the SARS-CoV-2 genomes in the coming months. This pointed out that many countries had multiple entries for this virus that could be claimed. Thus in the deduced network demonstrated transmission routes in different countries.

The study of mutations accumulation over time showed a higher number of mutations in the middle of the outbreak (end of January). At the same time, an increase in the number of mutations in early April was also observed. The first mutations to appear were mainly located in the intergenic region linked to the nucleocapsid phosphoprotein and the orf8 protein. The T265I, D614G and L84S hotspot mutations located in orf1ab and Spike proteins respectively were introduced into the virus for the first time in late February (**Fig 4B**).

### Phylogeographical analysis of SARS-CoV-2 genomes

The phylogenetic tree based on the whole genome alignment demonstrates that SARS-CoV-2 is wildly disseminated across distinct geographical location. The results showed that several strains are closely related even though they belong to different countries. Which indicate likely transfer events and identify routes for geographical dissemination.

Phylogenetic trees based on full-genome sequences deposited and available at GISAID and NCBI revealed the diversification, and the clustering of genomes into groups, based on the genetic variants. The phylogenetic analysis revealed two main clades C1 and C2; the original clade C1 harboring the mutation F3606L and starting since the beginning of the pandemic contains mainly Chinese strains from Dec to mid-Feb. After this period, the clade has emerged in other countries all over the globe. C1 is also composed of two subclades, SCB 1 sharig the mutation G251V (ORF3a) first identified in strains from china and further emerged in European strains, such as England and Iceland. The second subclade SCB2 also stared in China at the beginning of Jan and harbored the mutation L84S (ORF8). Following the first appearance it started emerging in other European countries mainly in Spain, this clade has also emerged in the USA in mid-Jan and gives birth to a new cluster containing 444 strains all sharing a C17747T mutation (Leu5828Leu, ORF1ab) starting from mid-Feb. Strains from the second clad C2 shared the spike mutation D614G (S) and harbored three subclades, this clade started in shanghai end of Jan. However, it contains mainly strain from Europe and North America. The first subcluster SCB3 harbored strain sharing two mutation R203K (N) and G204R (N) harboring largely strains from Europe and some strains from North Africa (France and USA). The second subcluster SCB4 harbored strain from Europe with the Q57H (ORF3a) mutation, these clusters started in France and Netherland during mid-Feb. genomes of Countries originated from Australia, South America, and Africa are scattered through the entire tree (**Fig 5**).

For phylogenetic tree (http://covid-19.medbiotech.ma) showed multiple introduction dates of the virus inside the USA with the first haplotype introduced related to the second epidemic wave in China.

**Fig 6:**
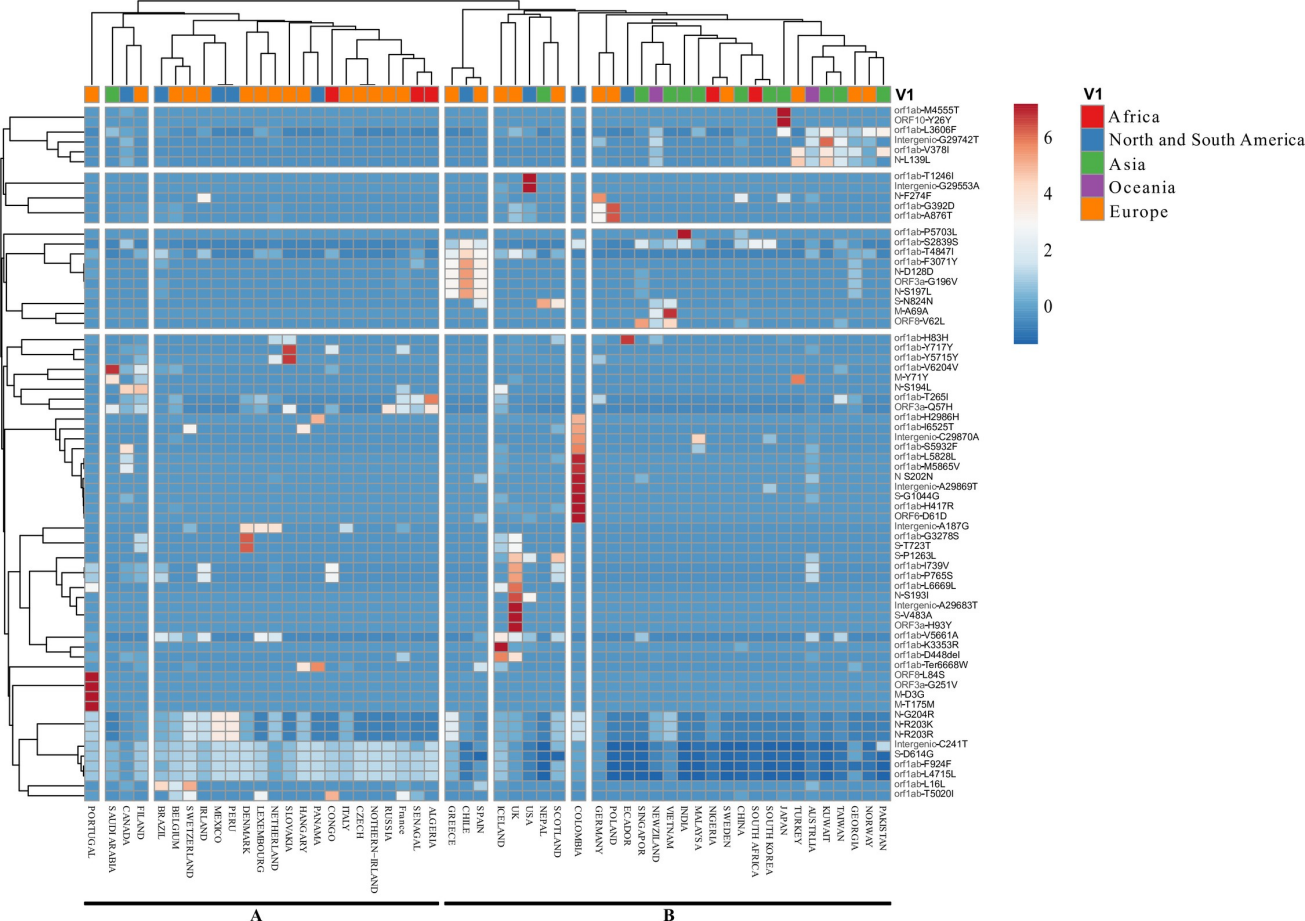
Heatmap showing the correlation between mutations and the geographic distribution of the genomes analyzed. The correlation was applied to a data set of 68 most recurrent mutations with different distribution in all 55 countries divided into two distinct cluster A and B. The color scale indicates the significance of correlation with blue and orange colors indicating the highest and lowest correlation. The red, yellow and orange colors in the horizontal bar represent the continent of origin. Abbreviations: S, spike; M, membrane protein; N, nucleocapsid protein.

Using correlation analysis between most recurrent mutations and countries distribution (**Fig 6**). We observed that most recurrent mutations clusters could be divided into four groups; the bigger cluster compromised nine mutations from the ten hotspots, while the first cluster harbored only the orf1ab mutation L3606F.

Meanwhile, geo clustering by geographic location showed two distinct clusters (**Fig 6**), cluster A grouping countries from Europe with those from America and Africa. However, Asia was only represented by Saudi Arabia. Cluster B in the other hand contained the majority of countries from the Asian and Australian continents. It is also harboring a sub-cluster containing the UK, USA, and Ireland which was previously demonstrated to contain a high number of mutations.

On the other hand, mutations as V378I and L3606F (in orfab1), 29742 C>T (intergenic), L139L in (in nucleocapside) were mainly correlated with Pakistan, Norway, Georgia, Taiwan, Kuwait, Australia, and Turkey while (S2839S, F3071Y and T4847I), D128D and G196V mutations in orf1ab, nucleocapsid, ORF3a, respectively, were mainly present in Spain, Chile, and Greece. However, cluster harboring D614G (in spike), F924F (in orf1ab), and L4715L (in orf1ab) mutations, showed no correlation and were scatted through all countries especially those from Europe. A high correlation with a specific mutation was observed within Portugal, Saudi Arabia, Slovakia, Iceland, UK, USA, Colombia, Ecuador, Vietnam, Japan genomes.

### Selective pressure analysis

Selective pressure on orf1ab, gene harbored a high rate of mutations and on the Spike gene, indicated a single alignment-wide ω ratio of 0.571391 and 0.75951 for spike and or1ab, respectively. Most sites for both genes had ω <1 values, indicating purifying selection. In orf1ab, we estimated eight sites under negative selection pressure (696, 1171, 2923, 3003, 3715, 5221, 5704 and 6267) and three sites under positive selection pressure (1473, 2244 and 3090). For spike, we found seven sites under negative selection pressure (215, 474, 541, 809, 820, 921 and 1044), and only one site under negative selection pressure (**Table 1**). None of the hotspot mutations was identified under negative selecion, this is moslty due sampling size and early date of sample collection.

**Table 1:** Selective pressure analysis on the spike and orf1ab genes of SARS-CoV-2.

Genes	Ω	FEL method		MEME method	SLAC method		FUBAR method	
**Spike**	0.571391	**PS**	**NS**	**PS**	**PS**	**NS**	**PS**	**NS**
		**-**	Codons 215, 474, 809, 820, 921, 1044	**-**	**-**	**-**	Codon 5	Codons 215,
								474, 541, 809, 820, 921, 1044
**orf1ab**	0.75951	**PS**	**NS**	**PS**	**PS**	**NS**	**PS**	**NS**
		Codon 2244	Codons	Codon2244	**-**	**-**	Codons 1473,	**-**
			1171, 2923, 3003, 3715,					
							2244, 3090	
			5221, 5704,					
			6267, 6961					

**Fig 7:**
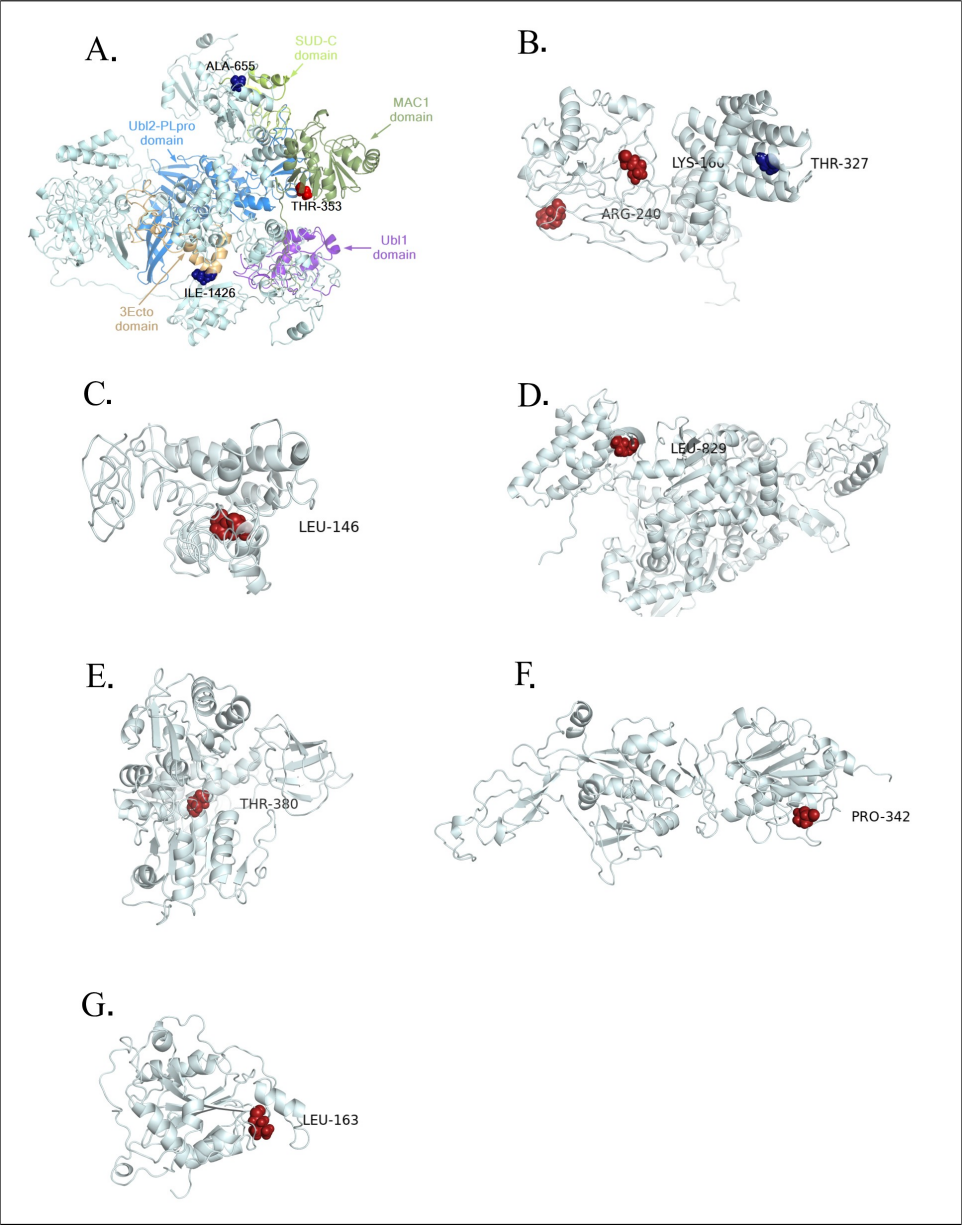
Structural view of selective pressure in orf1ab gene. The residue under the positive and negative selection is highlighted in blue and red respectively. The modeling of orf1ab non-structural proteins (nsp3, nsp4, nsp6, nsp12, nsp13, nsp14, and nsp16) harboring residues under pressure selection was produced using CI-TASSER. A. The nsp3 domains MAC1, Ubl1, Ubl2-PLpro, and SUD-C are color-coded in the 3D representation. The residues Ile-1426 and Ala-655 under negative selection are located respectively on 3Eco and SUD-C domains while Thr-353 residue under positive selection is shown on the MAC1 domain. Likewise, B, C, D, E, F, and G illustrating 3D representation of the nsp4, nsp6, nsp12, nsp13, nsp14 and nsp 16 proteins, respectively.

**Fig 8:**
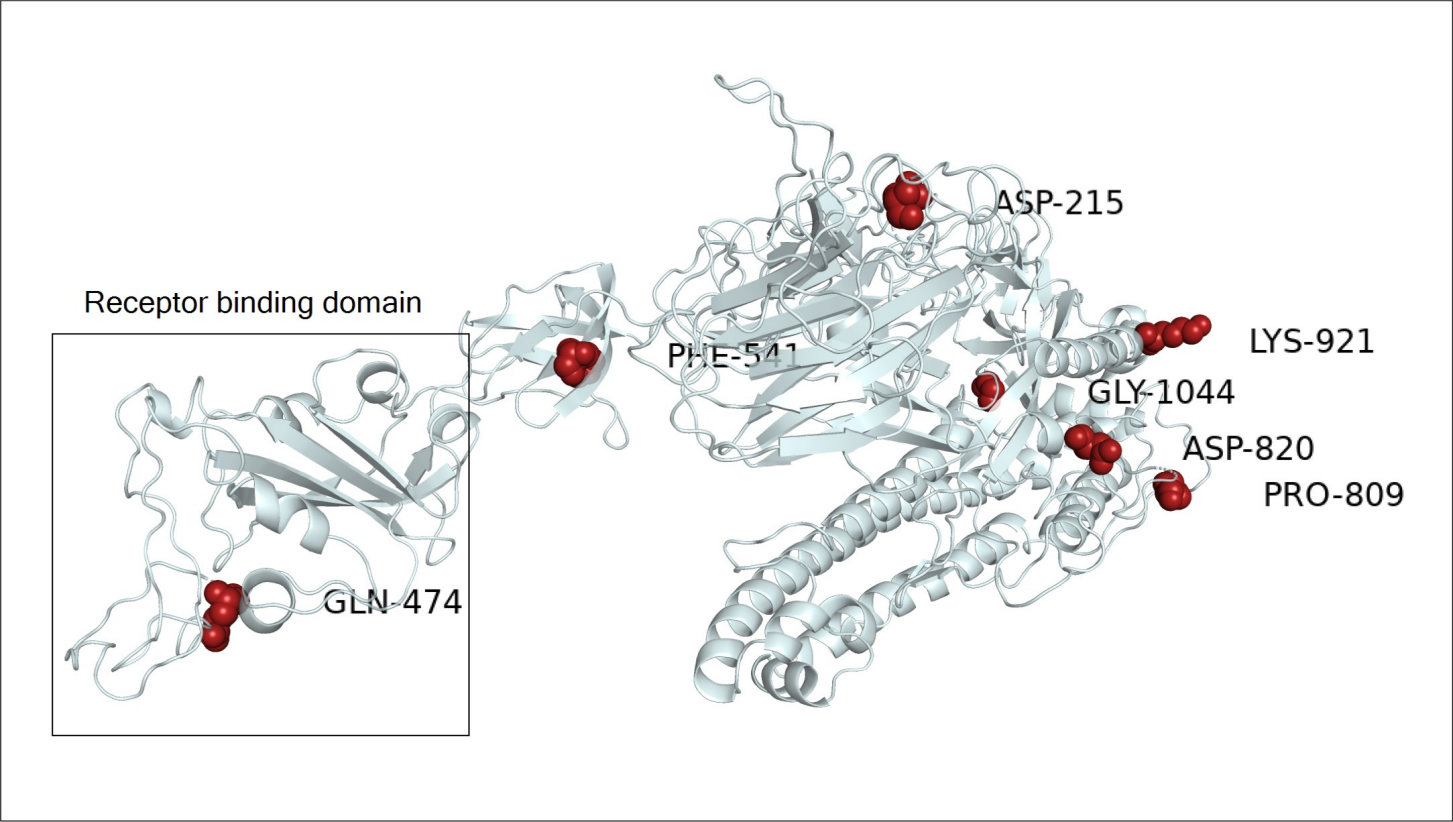
Structural view of selective pressure in spike gene. The negatively selected site in spike protein is highlighted in red. The only amino acid residue selected negatively on the receptor-binding domain corresponds to GLN-474. The cryo-EM structure with PDB id 6VSB was used as a model for the spike gene in its prefusion conformation.

The modelling results of orf1ab showed that the sites with positive selections were distributed in nsp3 and nsp4, while the negatively selected codons were located in nsp3, nsp4, nsp6, nsp12, nsp13, nsp14 and nsp16 (**Fig 7**). In spike, the only negatively selected residue was observed in the RBD region (**Fig 8**).

### Inter and intra-specific pan-genome analysis

**Fig 9:**
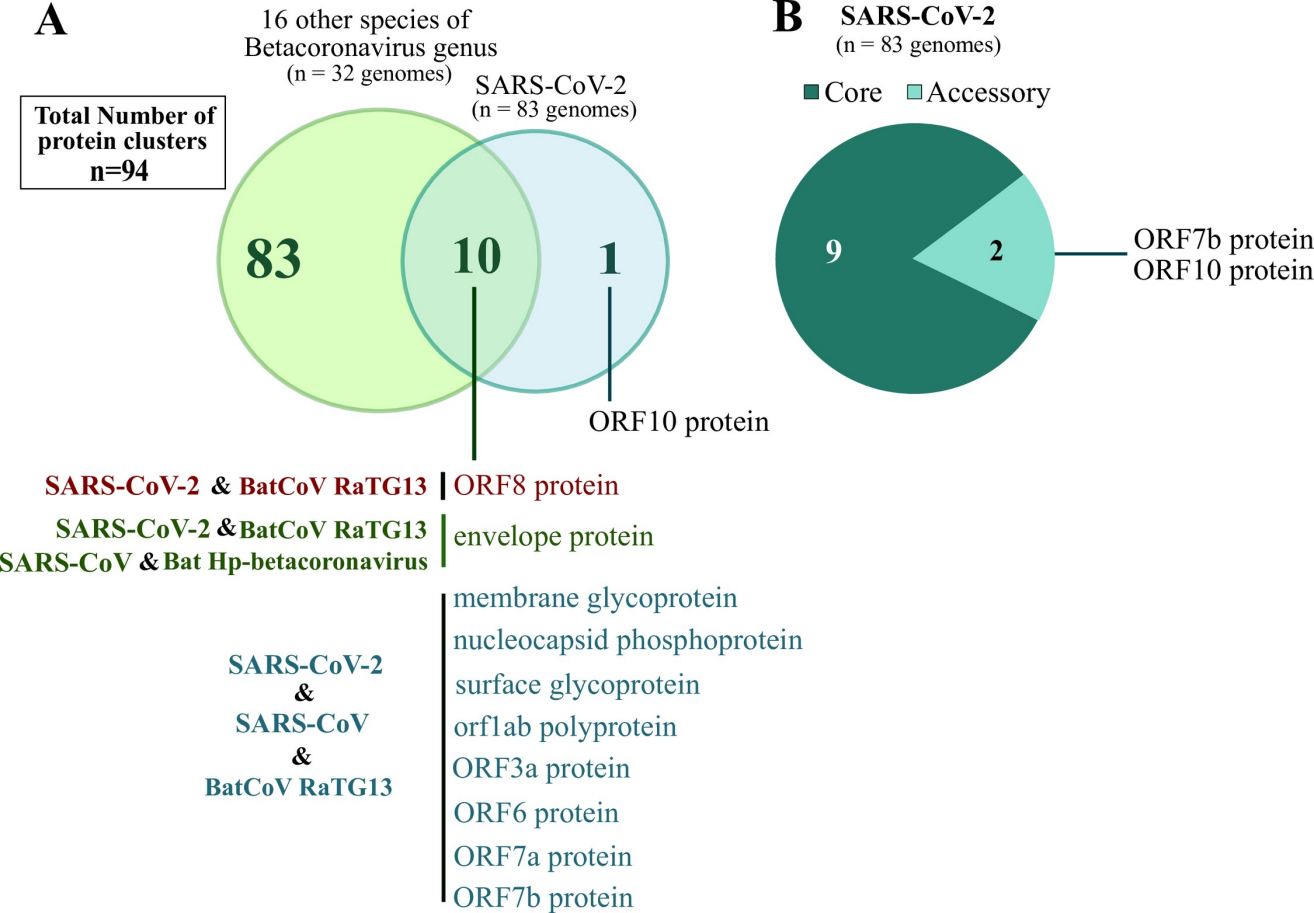
Pangenome construction of different strains belonging to the genus *Betacoronavirus*. A. The Venn diagram represents the shared and unique proteins of SARS-CoV-2 compared to the 16 species of the genus *Betacoronavirus*. B. The pie diagram showing the core (present in all strains) and accessory proteins (not present in all strains) at the intragenomic scale of SARS-CoV-2.

In order to highlight the proteins shared between SARS-CoV-2 and other species of the genus *Betacoronavirus*, Likewise, the proteins shared on the intra-genomic scale of SARS-CoV-2, we have constructed a pan-genome by clustering the sets of proteins encoded in 115 genomes distributed in 17 species, including 83 genomes belonging to SARS-Cov-2 (**S3 Table**). A total of 1,148 proteins were grouped into a pangenome of 94 orthologous protein clusters. Among them, ten protein clusters were shared between SARS-CoV-2 and only three species of the genus *Betacoronavirus*, including; BatCoV RaTG13, SARS-CoV and Bat Hp-betacoronavirus / Zhejiang2013. The BatCoV RaTG13 genome had more orthologous proteins shared with SARS-CoV-2, followed by SARS-CoV with ten and nine orthologous proteins, respectively (**Fig 9A**). It is interesting to note that among all the strains used of *Betacoronavirus*, the protein ORF8 was found in orthology only between SARS-RATG13 and SARS-CoV-2. In addition, the ORF10 protein was found as a singleton for SARS-CoV-2 genomes.

On the other hand, the analysis of the pangenome at the intra-genomic scale of 83 isolates of SARS-CoV-2 (**Fig 9B**), showed that ORF7b and ORF10 were two accessory genome (proteins variable between genomes) in SARS-CoV- 2 genomes, while the other proteins belonged to the core genome (present in all strains) of SARS-CoV-2.

## Discussion

The rate of mutations results in viral evolution and variability in the genome, thus allowing viruses to escape host immunity, as well as drugs [21]. Initial published data suggests that SARS-CoV-2 is genetically stable [22] which may increase the effectiveness of vaccines under development. The study on the genomic variation of SARS‐CoV‐2 is very important for the investigation of pathogenesis, disease course, prevention, and treatment of SARS‐CoV‐2 infection. In this study, we characterized the genetic variations in a large population of SARS-CoV-2 genomes. Our results showed a diversity of mutations detected with different frequencies. Overall, more than 500 non-synonymous mutations in SARS-CoV-2 genomes have been identified. The orf1ab gene having more than half the size of the SARS-CoV-2 genome and is divided into 16 nsp (nsp1-nsp16) [23]. We found more than half of recurrent mutations in orf1ab, and a high mutation rate in nsp3, nsp12 and nsp2, with 124, 57 and 46, respectively. Nsp2 and nsp3 were both essential for correcting viral replication errors [24]. Thus, recent studies have suggested that mutations falling in the endosome—associated—protein—like domain of the nsp2, could explain why this virus is more contagious than SARS [25].

The replication enzymes nsp12 to nsp16 have been reported as antiviral targets for SARS-CoV [26]. In the SARS-CoV-2 genomes, we found that nsp12 to nsp15 harbored nine recurrent non-synonymous mutations. Among them, eight identified as new mutations, including three in nsp12-RNA-dependent RNA polymerase (M4555T, T4847I and T5020I), three in nsp13-Helicase (V5661A, P5703L and M5865V) and two in nsp15-EndoRNAse (I6525T and Ter6668W). However, these new mutations must be taken into account when developing a vaccine using the orf1ab protein sequences as a therapeutic target.

A high number of mutations were identified in the spike protein, an important determinant in pathogenicity that allows the virion attachment to the cell membrane by interacting with the host ACE2 (Angiotensin-converting enzyme 2) receptor [27]. Among all the frequent mutations in this protein, the V483A mutation has been identified in this receptor and found mainly in SARS-CoV-2 genomes isolated from USA. This result is consistent with the study of Junxian et al. [28]. Eight stains from china, USA and France harbored V367F mutation previously described to enhance the affinity with ACE2 receptor [28].

Interestingly, ten hyper variable genomic hotspots with high frequencies of mutated allel detected. Among them, position 11083 (L3606F) detected in nsp6, this protein works with nsp3 and nsp4 by forming double-membrane vesicles and convoluted membranes involved in viral replication [29]. Besides, three positions were previously reported by Pachetti et al. (2020) [21], of which the two positions 17858 (M5865V) and 18060 (S5932F) in orf1ab, and 28881 (R203K) in nucleocapside. Moreover, intraspecies pangenome analysis of SARS- CoV-2 showed that the six of the genes harboring hotspot mutations belong to the core genome.

Thus, under normal circumstances genomic variation increase the viruses spread and pathogenicity. This happens when the virus accumulated mutation enabling its virulence potential [30]. Genomic comparison of the studied population allowed us to gain insights into virus mutations occurrence over time and within different geographic areas. In the SARS-CoV virus, the SNPs distribution is not random, and it is more dominant in critical genes for the virus [21, 31]. Our results confirmed what was previously described and elucidate the presence of numerous hotspot mutations. Besides, co-occurrence mutations were also common in different countries all along with singleton mutations. In the case of the China, the singleton mutations are driven by the single group that diverged differently due to the environment, the host, and the number of generations. These mutations are due to the low fidelity of reverse transcriptase [30, 32].

China, US, France and Malaysia contain a high number of specific mutations which may be the cause of a rapid transmission, especially in the US. These specific mutations may also be correlated with the critical condition in US and France.

The clustering of these genomes revealed the spread of clades to diverse geographical regions. We observed an increase of mutations over time following the first dissemination event from China. Specific haplotypes were also predominant to a geographical location, especially in the China. This study opens up new perspectives to determine whether one of these frequent mutations will lead to biological differences and their correlation with different mortality rates.

Among the seven nsp of or1ab hosting sites under selective pressure, only nsp3 and nsp4 contains both residues under positive and negative selection. The modelling of nsp3 domains shows that only the negative selection site 1171 (Thr- 353), was located at the conserved macro domain Mac1 (previously X or ADP-ribose 1" phosphatase) [33]. This domain has been previously shown to be dispensable for RNA replication in the context of a SARS-CoV replicon [34]. However, it could counteract the host's innate immune response [35]. It was proposed that the 3Ecto luminal domain of nsp3 interacts with the large luminal domain of nsp4 (residues 112–164) to ''zipper'' the endoplasmic reticulum (ER) membrane and induce discrete membrane formations as an important step in the generation of ER-origin viral replication organelles [36, 37]. As we have shown previously by the FEL, MEME and FUBAR methods, the orf1ab 2244 site coding for ILE-1426 is under positive selection pressure and since it is located on the luminal 3ecto domain of the nsp3 protein, this can be explained by a possible host influence on the virus in this domain. The results of selective pressure analysis revealed the presence of several negatively selected residues, one of which is located at the receptor-binding domain (GLN-474) and which is known by its interaction with the GLN24 residue of the human ACE2 receptor [38]. While this study allowed the identification of several site under selective pressure we would like to point that the size of the dataset could be a potential limitation for this type of study, therefore didn’t allow the identification of other site under selective pressure. Hence the need for a larger dataset. In general, it is well-known that negatively selected sites could indicate a functional constraint and could be useful for drug or vaccine target design, given their conserved nature and therefore less likely to change [39].

## Conclusion

The SARS-CoV-2 pandemic has caused a very large impact on health and economy worldwide. Therefore, understanding genetic diversity and virus evolution become a priority in the fight against the disease. Our results show several molecular facets of the relevance of this virus. We have shown that recurrent mutations are distributed mainly in six SARS-CoV-2 genes with variable mutated allele frequencies. We were able to highlight an increase in mutations accumulation overtime and revealed the existence of three major clades in various geographic regions. Finally, the study allowed us to identify specific haplotypes by geographic location and provides a list of sites under selective pressure that could serve as an interesting avenue for future studies.

## Supporting information

S1 Table Accession number, isolation date and origins of the complete SARS-CoV-2 genomes downloaded from the GISAID and NCBI database. (XLSX)

S2 Table GenBank accession numbers for orf1ab and spike genes downloaded from NCBI and used for selective pressure analysis. (XLSX)

S3 Table Clusters of orthologous proteins identified in the pangenome of 115 proteomes of the genus *Betacoronavirus*. (XLSX)

S4 Table Details about the singleton mutations identified in this study. (XLSX)

S5 Table Normalized data for singleton mutations identified in this study. (XLSX)
